# The levels of monoamine neurotransmitters and measures of mental and emotional health in HCV patients treated with ledipasvir (LDV) and sofosbuvir (SOF) with or without ribavirin (RBV)

**DOI:** 10.1097/MD.0000000000005066

**Published:** 2016-11-18

**Authors:** Pegah Golabi, Elzafir Elsheikh, Azza Karrar, James M. Estep, Issah Younossi, Maria Stepanova, Lynn Gerber, Zobair M. Younossi

**Affiliations:** a Betty and Guy Beatty Center for Integrated Research, Inova Health System, Falls Church, VA; b Center for Liver Disease, Department of Medicine, Inova Fairfax Hospital, Falls Church, VA.

**Keywords:** cytokine, emotional health, hepatitis C, mental health, neurotransmitter

## Abstract

Mental and emotional health (MEH) impairment is commonly encountered in hepatitis C patients. Although the exact mechanism remains unknown, alterations in neurotransmitter and cytokine levels maybe associated with hepatitis C virus (HCV)-related MEH issues.

The aim of the study was to assess association of serum biomarkers with self-reports of MEH in HCV patients before treatment and after achieving sustained virologic response (SVR).

The HCV genotype-1-infected patients who achieved SVR at 12 weeks after treatment with ledipasvir (LDV)/sofosbuvir (SOF) ± ribavirin (RBV) were selected. Frozen serum samples from baseline, end of treatment (EOT), and posttreatment week 4 (PTW4) were used to assay 16 cytokines and monoamine neurotransmitters. Validated self-reports were used to assess MEH.

Hundred patients were evaluated. Mean age was 53 years (57% male, 86% white). Compared with baseline, emotional well-being and emotional health significantly increased by EOT, and role emotional, emotional well-being, and emotional health significantly increased at PTW4 in the RBV-containing arm (*P* < 0.05). In patients taking LDV/SOF + RBV, serotonin levels were significantly decreased at PTW4 compared with baseline (*P* = 0.046). Compared with baseline, there were significant decreases in interleukin (IL)-10 levels at EOT and PTW4 in both treatment groups. The changes in IL-8 also differed significantly between LDV/SOF + RBV and LDV/SOF groups (*P* < 0.05). Changes in dopamine and tryptophan levels at EOT correlated with increasing emotional health scores, whereas changes in monocyte chemoattractant protein-1 at EOT and IL-8 at PTW4 correlated with increasing mental health scores. The neurotransmitters and cytokines were found to be independent predictors of MEH scores in multiple regression analysis.

Cytokine and neurotransmitter changes are associated with mental and emotional health. Patient-reported outcome scores change during and after treatment.

## Abstract 2

Supplemental Digital Content is available in the text

## 1 Introduction

Hepatitis C virus (HCV) is a major cause of chronic liver disease worldwide.^[1]^ More than 4 million people in the United States and nearly 185 million patients globally are infected with HCV.^[2,3]^ HCV causes a systemic infection with both hepatic manifestations (eg, cirrhosis and hepatocellular carcinoma) and extrahepatic manifestations involving multiple other organ systems (eg, integumentary, ocular, muscular, skeletal, nervous, endocrine, cardiovascular, respiratory, and urinary systems).^[4–6]^ Chronic HCV infection has been shown to impair patient-reported outcomes (PROs), such as health-related quality of life (HRQOL) and work productivity, and also deficits in attention, concentration, memory, mood, and information processing speed, collectively referred as “brain fog.”^[7–10]^

Previous studies have revealed that patients with chronic HCV infection have a higher prevalence of mental illness than the general population.^[11,12]^ These abnormalities are present in more than 50% of patients with HCV infection, including depression and anxiety disorders, which are present in nearly 30% of HCV-infected patients.^[13,14]^ Additionally, some specific patient populations such as intravenous drug users have increased risk for mental disorders.^[15–17]^ Although the relatively high prevalence of mental disorders among HCV patients may be related to patients’ characteristics, other virus-related mechanisms may also play an important role. In this context, it is important to remember that HCV is a member of the Flaviviridae family, and can replicate in the central nervous system (CNS).^[9,18,19]^ Recent data have suggested that defective serotoninergic and dopaminergic neurotransmission in the CNS, and also cytotoxic effects caused by circulating inflammatory cytokines, play key roles in CNS dysfunction caused by HCV infection.^[19–21]^ Furthermore, changes in tryptophan metabolism and the levels of interleukin (IL)-6, soluble IL-2 receptor, IL-8, IL-10, and tumor necrosis alpha (TNF-α) may additionally be engaged in the development of HCV-associated CNS abnormalities.^[8,9,22,23]^ On the contrary, regardless of HCV infection, the monoamine hypothesis has been an early milestone in the field of depression, in which depression is postulated to reflect a deficiency or imbalance in noradrenaline or serotonin.^[24]^ Additionally, associations between inflammatory markers (such as IL-6, IL-1-beta, and TNF-α) and depression, fatigue, cognitive dysfunction, and impaired sleep have been described previously.^[25–27]^

Despite these data, the exact pathophysiological mechanisms of neuropsychiatric disorders in patients with HCV remain poorly understood. Furthermore, the relationships among pathophysiological abnormalities and symptoms are not well described. In the past few years, multiple studies have evaluated the response of neuropsychiatric disorders to HCV eradication with interferon and ribavirin therapy, and also measuring changes in serum levels of certain neurotransmitters and cytokines.^[28–35]^ In contrast, data are limited with the new direct-acting antiviral (DAA) regimens for HCV.

In this study, our aim was to assess the association between serum levels of selected neurotransmitters and cytokines with self-reports of mental and emotional health (MEH), as measured by validated instruments for assessment of PROs, in patients with chronic HCV infection who achieved sustained virologic response (SVR) with ledipasvir (LDV)/sofosbuvir (SOF) with or without ribavirin (RBV).

## 2 Method

This is a retrospective study utilizing the data from ION-1 clinical trial. The study sample was selected from patients with chronic hepatitis C infection who participated in ION-1 clinical trial. ION-1 was a phase 3 clinical trial that enrolled treatment-naive HCV genotype 1 infected patients to receive a fixed-dose combination tablet containing 90 mg of LDV and 400 mg of SOF once daily with or without weight-based RBV (1000 or 1200 mg/d).^[36]^ For this study, we selected 100 patients who achieved a SVR at posttreatment follow-up week 12 (SVR-12) and had no missing information on the key components of this study. Serum samples had been collected and frozen at 3 time points: baseline, end of treatment (EOT), and posttreatment follow-up week 4 (PTW4). For each subject, medical history, including history of psychiatric disorders, was collected at screening.

### 2.1 Neurotransmitter measurement

Sera concentrations of dopamine (pg/mL), norepinephrine (pg/mL), tryptophan (nmol/mL) (Abbexa Ltd, Cambridge, United Kingdom), and serotonin (ng/mL), (Enzo life Science, Farmingdale, NY) were measured by enzyme-linked immunosorbent assay as per the manufacturer's instructions. Briefly, 50 μL diluted sera/standards were added onto the precoated plates according to the template. The plates were then washed, 50 μL horseradish peroxidase-conjugated antibodies were added to each well, and the plates were then incubated at 37°C for 30 minutes. After washing, 50 μL of the tetramethylbenzidine (TMB) substrate A was added followed by 50 μL of TMB substrate B. The plates were then incubated in darkness at 37°C for 15 minutes followed by stopping the reaction with 50 μL of stop solution. The optical density absorbance of the plates is read at 450 nm in a microplate reader for 15 minutes. The human neurotransmitter concentrations were calculated using a 5-parameter logistic curve. For serotonin measurement, 150 μL of assay buffer, and 100 μL each of the standard and samples were added to precoated wells. This was followed by the addition of 50 μL of conjugate and 50 μL of antibodies to the wells according to the pan. After incubation for 2 hours on a plate shaker at room temperature, plates were washed and 5 μL of the conjugate and 200 μl of the substrate solution were added to the plates and incubated for 1 hour at room temperature with shaking. The reaction was stopped by the addition of 50 μL of Stop solution to each well. The plates were read at 405 nm. The concentration of serotonin for each patient was determined by interpolation.

### 2.2 Assessment of mental and emotional health

A number of PRO measures were administered by questionnaire to the study participants at the 3 study time points. Four MEH domains from the below mentioned and extensively validated instruments were utilized in this study.

The Short Form 36 (SF-36) questionnaire is a generic instrument commonly used to assess health-related quality of life (HRQOL) of patients and is composed of the following 8 domains: physical functioning, role physical, bodily pain, general health, vitality, social functioning, role emotional (RE), and mental health (MH). In this study, we focused on the RE and MH domains of the SF-36 questionnaire, which range from 0 to 100, with greater values indicating better emotional and mental health.^[37]^

The Chronic Liver Disease Questionnaire—Hepatitis C Virus (CLDQ-HCV) is a validated disease-specific PRO instrument which has been developed to capture health impairment features most frequently seen in patients with chronic HCV infection and composed of the following 4 domains: activity and energy, emotional, worry, and systemic. In this study, the emotional health (EMM) component of CLDQ-HCV was used, which ranged from 1 to 7.^[38]^

Functional Assessment of Chronic Illness Therapy—Fatigue (FACIT-F) questionnaire is another validated instrument that consists of the following components: physical, emotional, social, and functional well-being domains, and also a fatigue subscale.^[39]^ For this analysis, we utilized the emotional well-being (EWB) domain of FACIT-F, which ranges from 0 to 24, with greater scores indicating better health.

### 2.3 Statistical analysis

Univariate analysis was performed to compare the treatment groups LDV/SOF versus LDV/SOF + RBV using a chi-square test for categorical outcomes (such as sex) and Mann–Whitney nonparametric test for continuous outcomes (such as an MEH score or a cytokine level). The changes in MEH domain scores and cytokines and neurotransmitters from patient's own baseline levels were also calculated and compared with 0 using a nonparametric sign-rank test; the changes in neurotransmitters and cytokines were calculated relative to the respective baseline levels. Independent predictors of the 4 MEH outcomes were assessed by using multiple linear regressions. Correlations of MEH items were calculated using Spearman nonparametric method. All analyses were run in SAS 9.3 (SAS Institute, Cary, NC).

The original clinical trial was approved by the institutional review board or independent ethics committee at each participating clinical site and was conducted in compliance with Good Clinical Practice guidelines and local regulatory requirements. The study was approved by Inova Institutional Review Board.

## 3 Results

A total of 100 (50 LDV/SOF; 50 LDV/SOF + RBV) treatment-naive HCV genotype 1 infected subjects, who achieved SVR-12, were selected for this study. For each patient, pretreatment and posttreatment frozen serum samples were available. The mean age of the patients was 53 (±10) years; 57% were male and 86% were white. General characteristics of the study population, pretreatment medical histories of the patients, and treatment-related adverse events are shown in Table 1.

**Table 1:**
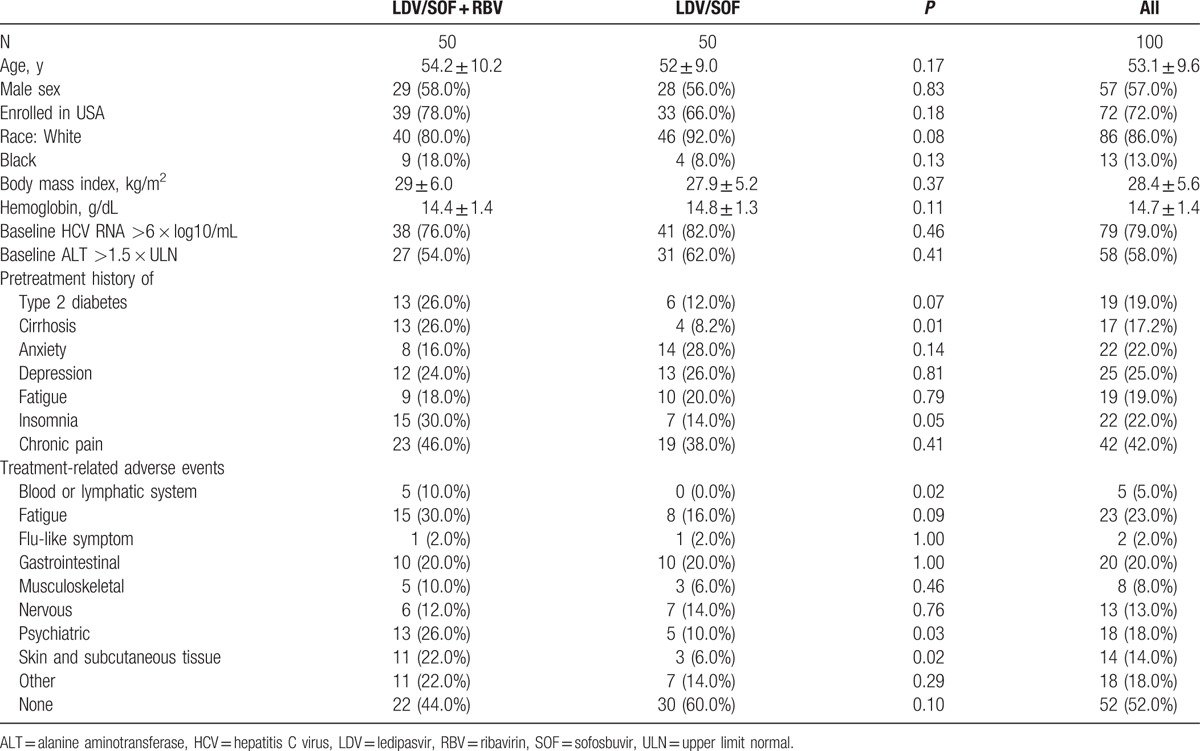
General characteristics of the study population at baseline.

### 3.1 Changes in mental and emotional health indicators

For both RBV-containing and RBV-free cohorts, viral suppression resulted in a significant increase in EWB and EMM scores at the end of treatment (*P* < 0.05) (Table 2). Also, compared with baseline levels, all MEH domain scores, except MH, significantly increased by PTW4 in the RBV-containing group, whereas only RE could not reach significance in the RBV-free arm (all *P* < 0.05). Among MEH indicators, the most impressive improvement was noted in EWB score at EOT and PTW4 for both cohorts (*P* < 0.001) (Table 2).

**Table 2:**
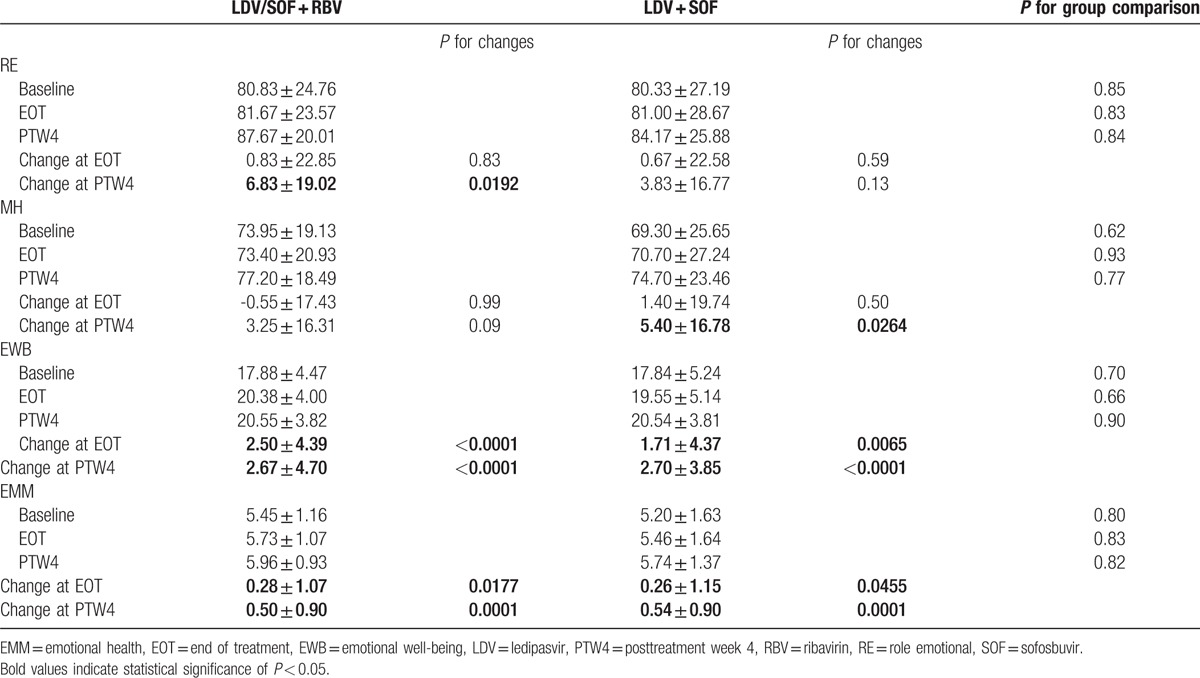
Mean baseline levels and changes in the MEH scores.

At baseline, there was no difference in MEH scores between the 2 treatment groups (Table 2). At the EOT, there was a statistically significant increase in EWB (on average, from 17.86 to 19.96 on a 0–24 scale; *P* < 0.001) and EMM (from 5.33 to 5.60 on a 1–7 scale; *P* = 0.002) domains in both the treatment groups. Also, when compared with baseline levels, there were significant increases at PTW4 in RE (80.83–87.67 on a 0–100 scale; *P* = 0.019, for RBV+ only), MH (69.30–74.70 on a 0–100 scale; *P* = 0.026, for RBV− only), EWB (17.86–20.54; *P* < 0.001, for both treatment groups), and EMM (5.33–5.85; *P* < 0.001, for both treatment groups).

### 3.2 Changes in neurotransmitter and cytokine levels

In patients receiving LDV/SOF + RBV, compared with baseline, serotonin levels significantly decreased at PTW4 (*P* = 0.046) (Table 3). Also, compared with baseline, the levels of granulocyte colony stimulating factor (GCSF) significantly increased by the end of treatment (*P* = 0.0018) and PTW4 (*P* = 0.009). In those receiving RBV-free LDV/SOF, serum IL-8 levels significantly increased at EOT in comparison with baseline levels (*P* = 0.047) (Table 3). Additionally, compared with baseline levels, serum IL-10 levels significantly decreased at EOT and PTW4 in both groups treated with or without RBV-containing regimens (all *P* < 0.001). Also, serum lactate dehydrogenase (LDH) levels significantly changed at EOT (*P* = 0.039) and PTW4 (*P* = 0.013) in RBV+ group and at EOT (*P* = 0.009) in RBV− group. Similarly, compared with baseline levels, serum platelet-derived growth factor (PDGF) levels significantly increased at EOT (*P* = 0.015 and *P* = 0.044, respectively) in both RBV+ and RBV− groups (Table 3).

**Table 3:**
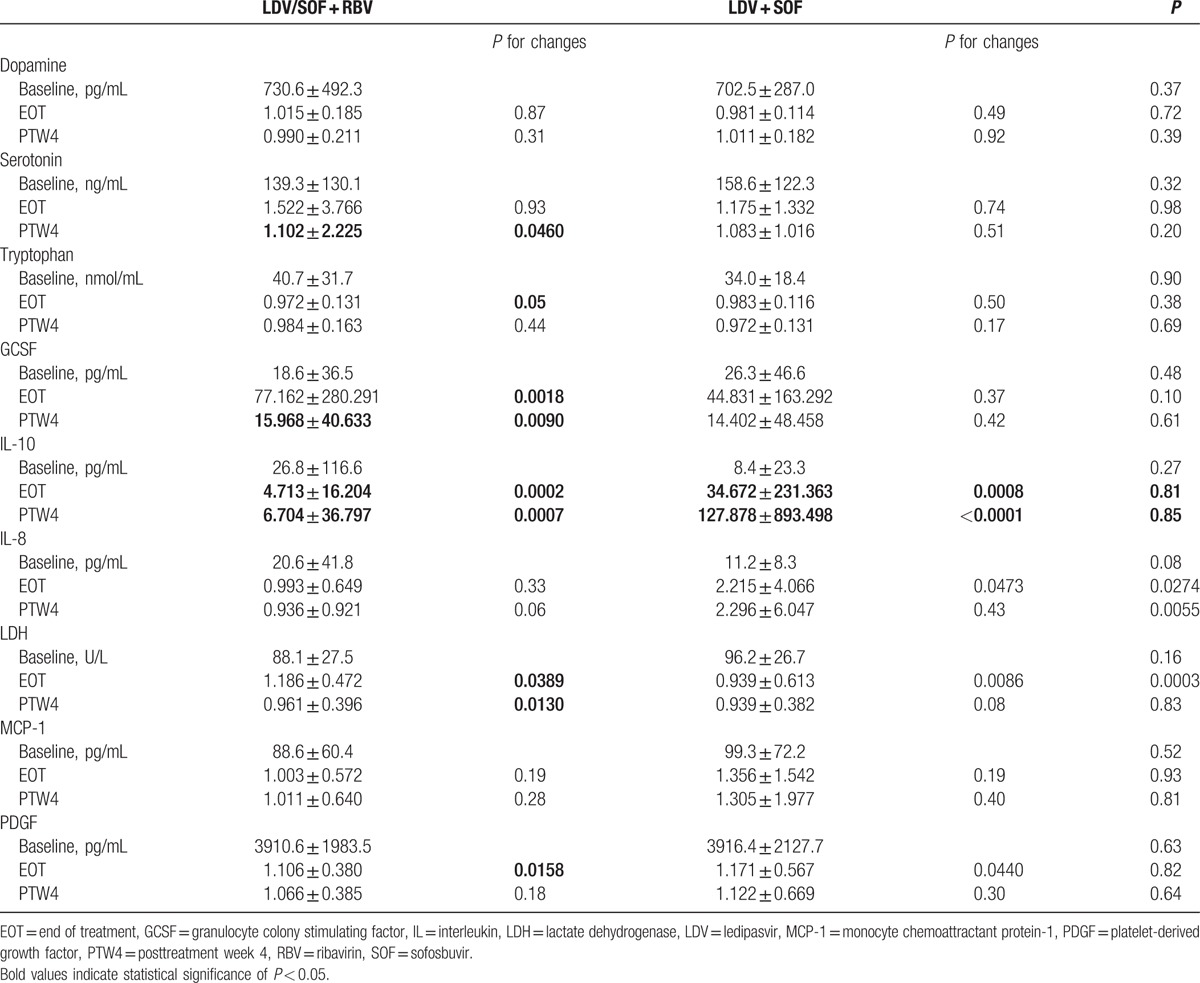
Baseline levels and relative-to-baseline changes of neurotransmitters and cytokines.

In comparison of RBV+ and RBV− groups, there were significant differences in the changes of IL-8 (both EOT and PTW4) and LDH concentration (EOT only) (*P* < 0.05) (Table 3).

### 3.3 Correlations between neurotransmitters and cytokines and MEH scores

At baseline (Supplementary Table 1), there was a significant and positive correlation of dopamine and RE score of SF-36 (*R* = 0.25, *P* = 0.011). Also, at baseline, there was a significantly negative correlation of cortisol and interferon (IFN)-γ with MH score of SF-36 (*R* = −0.19, *P* = 0.050 and *R* = −0.23, *P* = 0.021, respectively) and IFN-γ with EM score of CLDQ-HCV (*R* = −0.2594, *P* = .009).

By EOT (Supplementary Table 2), a change in the levels of tryptophan and dopamine was found to be positively correlated with a change in the RE score of SF-36 (*R* = 0.22, *P* = 0.025 and *R* = 0.22, *P* = 0.027, respectively). By EOT, changes in the levels of IL-8 and monocyte chemoattractant protein-1 (MCP-1) were also found to be positively correlated with a change in MH score of SF-36 (*R* = 0.19, *P* = 0.05 and *R* = 0.25, *P* = 0.010, respectively).

By PTW4 (Supplementary Table 2), a change in the level of PDGF was positively correlated with a change in EWB score (*R* = 0.24, *P* = 0.015), whereas a change in MH was positively correlated with a change in IL-8 (*R* = 0.23, *P* = 0.022).

### 3.4 Independent associations of MEH scores with neurotransmitters and cytokines

After adjustment for baseline history of depression, the baseline RE score was found to be independently and negatively associated with the levels of IL-8 and TNF-α (both *P* < 0.02) (Table 4). On the contrary, the MH and EWB scores were found to be independently and negatively associated with history of anxiety and the levels of cortisol and TNF-α (all *P* < 0.05). The EM score was found to be independently and negatively associated with history of depression and the level of TNF-α (all *P* < 0.05) (Table 4). The changes in the MEH scores by EOT were found to be positively associated with the respective changes in MCP-1 (RE and MH) and negatively with IL-8 (RE only). Finally, the PTW4 changes in the MEH scores were found to be positively associated with the changes in the level of PDGF (RE, EWB) and IL-1ra (EWB only), and negative with IL-8 (RE only) (Table 4).

**Table 4:**
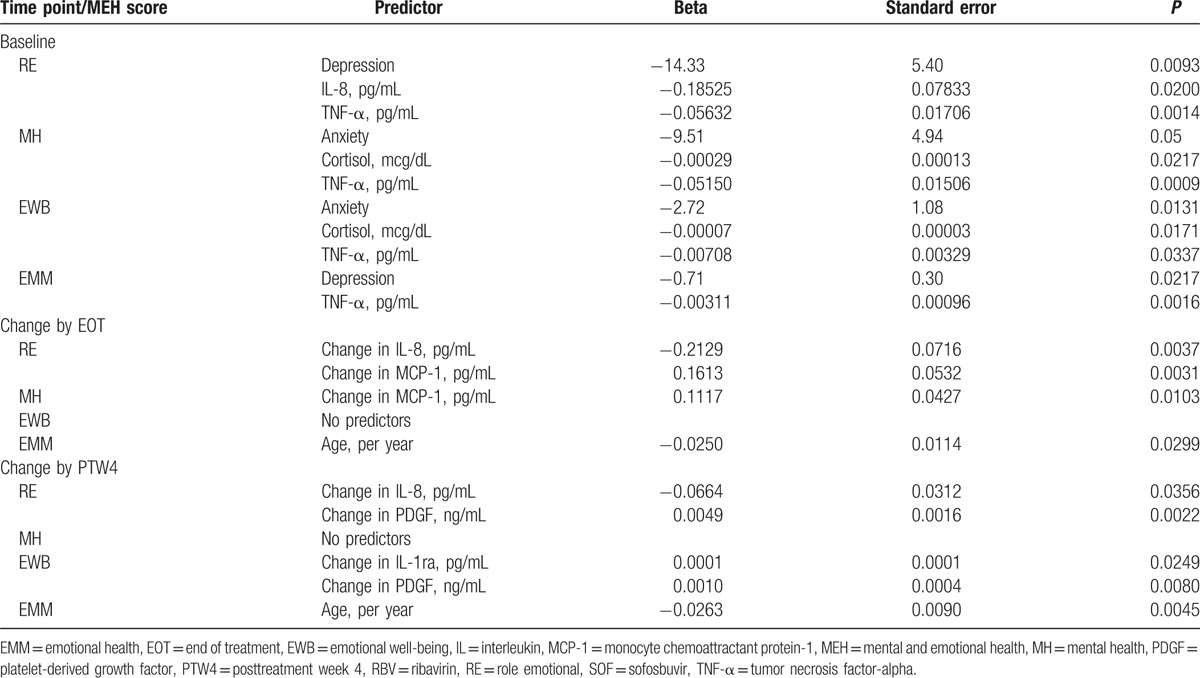
Independent predictors of MEH scores.

## 4 Discussion

In this study, the serum level of a number of neurotransmitters and cytokines of HCV-infected patients were compared at different time points before, during, and after treatment with LDV/SOF ± RBV, and correlated with the scores of different MEH PRO domain scores. To our knowledge, this is the first study to combine MEH PROs with the assessment of serum neurotransmitter and cytokine levels in patients with HCV who are cured with an IFN-free regimen.

Our data also provide some insight about the potential impact of RBV on MEH PROs and associated biomarkers. Our results show that serotonin levels can significantly increase after achieving SVR in patients receiving LDV/SOF + RBV, but not LDV/SOF alone. In contrast to the pattern seen for serotonin, no significant changes in the levels of cortisol, dopamine, norepinephrine, and tryptophan were seen in either treatment group. Additionally, only patients receiving treatment with LDV/SOF + RBV experienced a significant reduction in tryptophan and an increase in GCSF levels. Capuron et al^[40]^ and Cozzi et al^[41]^ have previously reported the association between decreased levels of tryptophan and the presence of depressive symptoms among HCV patients. Similarly, the correlation between increased levels of serotonin and decreased occurrence of depression and anxiety has been documented in previous studies.^[21,42–44]^ Weissenborn et al^[45,46]^ showed that the changes in midbrain serotoninergic and dopaminergic pathways were related with the increased anxiety and depression of patients with chronic HCV infection, and there was decreased binding capacity of these monoamine transporters in midbrain and striatal regions of patients with chronic HCV infection. The fact that changes occurred at the end of treatment only in those patients who were receiving RBV-containing regimens suggests that RBV may be a source of their elevation, rather than HCV infection. This may explain the association of RBV with psychologic issues and PRO impairments, even in the absence of IFN-based regimens.^[47]^ On the contrary, levels of IL-10, LDH, and PDGF changed significantly in both treatment groups. This suggests these changes are not likely to be influenced by RBV.

Another mechanism of CNS abnormality may be through the inflammatory pathway HCV causes in the brain. A study by Fletcher et al^[48]^ reported that human brain endothelial cells express functional receptors that support HCV entry and replication, and it was shown that HCV induces apoptosis in those cells, causing changes in the permeability of blood-brain barrier, activation of microglial cells, and diffusion of proinflammatory cytokines into the CNS. This local inflammatory response, which is mediated by IL-8 and TNF-α in HCV-infected microglial cells, results in the activation of the immune system, causing production of cytokines like IL-1, IL-6, IL-4, and TNF-α.^[49,50]^ One of the most striking findings of our study was that, serum IL-8 levels, and also the rates of changes between time points, demonstrated significant differences at the EOT and PTW4 time points, and that those differences were experienced differentially between RBV+ and RBV− groups. Indeed, the changes in IL-8 levels may be associated with feelings of illness or fatigue, as the rate of change in IL-8 was shown to be correlated with the mental health domain in our study.

Another important finding of our study was that the scores of all 4 PRO domains significantly improved after achieving SVR, with the most prominent increment in EWB of FACIT-F. Our findings support previous data about the MEH factors in patients with HCV.^[51–53]^ In a recent study among HCV patients who were treated with LDV/SOF with or without RBV, it was found that compared with baseline, the PRO scores, including EWB and most domains of CLDQ-HCV, significantly changed at the week 12 of treatment, and greater improvements were noted in RBV-free group.^[51]^ In our study, there was no significant difference between RBV+ and RBV− regimens, and in all 3 questionnaires, the scores of MEH domains significantly increased in the whole cohort.

This study also revealed that at baseline, dopamine levels significantly and positively correlated with RE scores of SF-36 in both treatment groups. Also, by EOT, a change in dopamine and tryptophan levels significantly and positively correlated with RE scores. These results were in accordance with a recent study among HCV patients treated with LDV/SOF. Using magnetic resonance spectroscopy, investigators have shown a negative correlation between basal ganglia myoinositol level, which serves as a secondary messenger in serotoninergic and dopaminergic pathways, and emotional domain of CLDQ-HCV at EOT.^[54]^

Finally, our study showed that the inflammatory marker, IFN-γ, at baseline, significantly and negatively correlated with MH scores of SF-36, and at EOT, a change in IL-8 levels positively correlated with MH scores. Furthermore, in comparison with the baseline levels, although MCP-1 levels decreased in RBV+ arm and increased in RBV− group, a change in MCP-1 level significantly and positively correlated with MH scores at EOT. However, it is not solely mental health that is affected with MCP-1 levels, as in a very recent study, Gerber et al^[55]^ showed that MCP-1 levels were associated with persistent fatigue after SVR-12 among HCV patients treated with LDV/SOF.

This study also has some limitations. First of all, the instruments used to evaluate MEH were subjective measurements that patients reported, although it can be assumed that individual differences were uniformly distributed in both groups. Another limitation can be a relatively short follow-up, where an elongated follow-up period might reflect the effect of achieving SVR more clearly. Also, it is noteworthy to mention that during the trial, patients were acknowledged about the changes in their viral load with medications, or the treatment success at the end of trial, which may affect their mood, but this is an inevitable factor.

In conclusion, our study showed that alteration of monoamine neurotransmitters and cytokine levels in HCV-infected patients may be associated with MEH. Also, achieving SVR with antiviral treatment is strongly associated with increases in MEH domains. More studies are needed to better understand the exact effect of HCV on neurotransmitter and inflammatory cytokine levels, and also MEH of these patients.

## Acknowledgments

The authors would like to thank Deena Hallaji, Mehmet Sayiner, MD, and Brian Lam, PA-C, for their great support during the formation of this manuscript.

## Supplementary Material

Supplemental Digital Content
