# Supplementary material for: The levels of monoamine neurotransmitters and measures of mental and emotional health in HCV patients treated with ledipasvir (LDV) and sofosbuvir (SOF) with or without ribavirin (RBV)
Source: Medicine (Baltimore). 2016 Nov 18;95(46):e5066. doi: 10.1097/MD.0000000000005066 (PMC5120894; doi:10.1097/MD.0000000000005066)
Supplement: Supplemental Digital Content [file medi-95-e5066-s001.docx]

**Supplementary Table 1:** Correlations (R) of MEH scores with neurotransmitter and cytokine levels at baseline.

|  | RE | | MH | | EWB | | EMM | |
| --- | --- | --- | --- | --- | --- | --- | --- | --- |
|  | R | p | R | p | R | p | R | p |
| Serotonin, ng/mL | 0.12 | 0.25 | 0.08 | 0.44 | **0.18** | **0.07** | 0.10 | 0.30 |
| Cortisol, mcg/dL | -0.12 | 0.23 | **-0.19** | **0.05** | -0.09 | 0.38 | -0.14 | 0.17 |
| Norepinephrine, pg/mL | -0.12 | 0.22 | -0.13 | 0.19 | -0.09 | 0.36 | -0.16 | 0.12 |
| Tryptophan, nmol/mL | 0.09 | 0.38 | -0.04 | 0.67 | -0.07 | 0.51 | -0.10 | 0.33 |
| Dopamine, pg/mL | **0.252** | **0.011** | **0.17** | **0.09** | 0.10 | 0.33 | 0.12 | 0.25 |
| IL1b, pg/mL | 0.03 | 0.75 | 0.02 | 0.85 | 0.07 | 0.51 | 0.04 | 0.66 |
| IL1ra, pg/mL | -0.03 | 0.76 | -0.12 | 0.22 | -0.03 | 0.78 | -0.11 | 0.29 |
| IL8, pg/mL | 0.00 | 0.97 | -0.10 | 0.31 | -0.04 | 0.69 | 0.01 | 0.93 |
| IL10, pg/mL | -0.10 | 0.31 | -0.14 | 0.16 | -0.06 | 0.55 | -0.15 | 0.14 |
| GCSF, pg/mL | -0.03 | 0.80 | 0.05 | 0.63 | 0.04 | 0.69 | -0.03 | 0.80 |
| IFN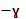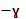, pg/mL | -0.12 | 0.23 | -0.23 | 0.02 | -0.14 | 0.16 | **-0.25** | **0.009** |
| MCP-1, pg/mL | 0.03 | 0.73 | -0.02 | 0.82 | 0.05 | 0.64 | 0.01 | 0.94 |
| PDGF, pg/mL | 0.04 | 0.72 | 0.09 | 0.38 | 0.07 | 0.46 | 0.12 | 0.24 |
| TNF-α, pg/mL | -0.09 | 0.38 | -0.03 | 0.73 | 0.06 | 0.56 | -0.02 | 0.88 |
| VEGF, pg/mL | 0.05 | 0.65 | 0.02 | 0.88 | 0.07 | 0.50 | -0.02 | 0.86 |
| LDH, U/L | 0.04 | 0.66 | -0.07 | 0.47 | -0.03 | 0.76 | -0.05 | 0.62 |

**Supplementary Table 2:** Correlations (R) of treatment-emergent MEH scores with changes in neurotransmitter and cytokine levels.

|  | RE | | MH | | EWB | | EMM | |
| --- | --- | --- | --- | --- | --- | --- | --- | --- |
|  | R | p | R | p | R | p | R | p |
| EOT change | | | | | | | | |
| Serotonin, ng/mL | -0.05 | 0.63 | -0.04 | 0.67 | 0.03 | 0.73 | -0.04 | 0.73 |
| Cortisol, mcg/dL | 0.05 | 0.62 | -0.02 | 0.82 | 0.09 | 0.40 | 0.00 | 0.99 |
| Norepinephrine, pg/mL | 0.08 | 0.45 | 0.12 | 0.25 | 0.15 | 0.13 | 0.19 | 0.06 |
| Tryptophan, nmol/mL | 0.20 | 0.048 | 0.16 | 0.12 | 0.16 | 0.11 | 0.14 | 0.15 |
| Dopamin, pg/mL | 0.21 | 0.036 | -0.11 | 0.27 | 0.02 | 0.88 | 0.12 | 0.25 |
| IL1b, pg/mL | 0.07 | 0.48 | 0.10 | 0.32 | 0.04 | 0.71 | 0.10 | 0.32 |
| IL1ra, pg/mL | 0.05 | 0.63 | 0.11 | 0.26 | 0.05 | 0.64 | 0.05 | 0.59 |
| IL8, pg/mL | 0.11 | 0.26 | 0.17 | 0.09 | 0.10 | 0.30 | 0.11 | 0.28 |
| IL10, pg/mL | -0.10 | 0.33 | 0.02 | 0.81 | -0.05 | 0.59 | -0.06 | 0.57 |
| GCSF, pg/mL | 0.11 | 0.29 | 0.13 | 0.19 | -0.01 | 0.96 | -0.03 | 0.77 |
| IFN-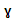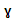, pg/mL | 0.08 | 0.40 | 0.05 | 0.64 | -0.04 | 0.71 | -0.09 | 0.39 |
| MCP-1, pg/mL | 0.16 | 0.10 | 0.27 | 0.007 | 0.07 | 0.47 | 0.14 | 0.16 |
| PDGF, pg/mL | -0.09 | 0.58 | 0.00 | 0.97 | 0.14 | 0.18 | 0.14 | 0.18 |
| TNF-α, pg/mL | 0.09 | 0.36 | 0.11 | 0.27 | 0.10 | 0.31 | 0.08 | 0.42 |
| VEGF, pg/mL | 0.07 | 0.49 | 0.14 | 0.18 | 0.04 | 0.72 | 0.12 | 0.23 |
| LDHconc, U/L | -0.09 | 0.36 | 0.03 | 0.74 | 0.00 | 0.98 | -0.11 | 0.30 |
| PTW4 change | | | | | | | | |
| Serotonin, ng/mL | -0.16 | 0.12 | -0.02 | 0.82 | -0.03 | 0.75 | -0.11 | 0.29 |
| Cortisol, mcg/dL | 0.02 | 0.84 | 0.03 | 0.76 | 0.09 | 0.39 | 0.05 | 0.65 |
| Norepinephrine, pg/mL | 0.08 | 0.44 | 0.08 | 0.42 | 0.12 | 0.23 | 0.05 | 0.64 |
| Tryptophan, nmol/mL | 0.17 | 0.10 | 0.18 | 0.07 | 0.15 | 0.14 | 0.12 | 0.24 |
| DOPAMIN, pg/mL | -0.06 | 0.54 | -0.08 | 0.41 | 0.09 | 0.37 | -0.03 | 0.77 |
| IL1b, pg/mL | 0.11 | 0.27 | 0.18 | 0.08 | 0.15 | 0.13 | 0.17 | 0.10 |
| IL1ra, pg/mL | 0.12 | 0.22 | 0.07 | 0.49 | 0.16 | 0.10 | -0.01 | 0.92 |
| IL8, pg/mL | 0.01 | 0.92 | 0.23 | 0.0223 | 0.16 | 0.12 | 0.17 | 0.09 |
| IL10, pg/mL | 0.19 | 0.06 | 0.12 | 0.25 | 0.11 | 0.27 | 0.14 | 0.17 |
| GCSF, pg/mL | 0.15 | 0.14 | 0.09 | 0.35 | 0.10 | 0.30 | 0.00 | 0.97 |
| IFN- ɣ, pg/mL | 0.08 | 0.43 | 0.04 | 0.72 | 0.06 | 0.56 | -0.10 | 0.35 |
| MCP-1, pg/mL | 0.09 | 0.35 | 0.10 | 0.32 | 0.07 | 0.50 | 0.08 | 0.45 |
| PDGF, pg/mL | 0.10 | 0.34 | 0.12 | 0.23 | 0.24 | 0.0148 | 0.05 | 0.62 |
| TNF-α, pg/mL | 0.16 | 0.13 | 0.08 | 0.42 | 0.10 | 0.31 | 0.04 | 0.72 |
| VEGF, pg/mL | -0.04 | 0.70 | 0.12 | 0.24 | 0.11 | 0.30 | -0.07 | 0.48 |
| LDH conc, U/L | 0.06 | 0.57 | 0.08 | 0.44 | -0.09 | 0.36 | 0.09 | 0.40 |
